# AI Technologies and International Relations: Do We Need New Analytical Frameworks?

**DOI:** 10.1080/03071847.2024.2392394

**Published:** 2024-08-29

**Authors:** Ingvild Bode

## Abstract

AI technologies are drawing increasing attention among international relations (IR) scholars. Ingvild Bode reviews this literature through considering, in particular, the extent to which such studies continue to use or expand on well-traded analytical frameworks. She finds that scholarship on AI in IR can look back at a longer-than-expected trajectory and centres on four key themes: the balance of power; disinformation; governance; and ethics. Much of this literature works with well-established IR conceptualisations, while studies across three emerging themes – (re)conceptualising technology, beyond the AI arms race, and unpacking relevant actors – push and expand established disciplinary frameworks. ◼

The study of AI technologies in international relations (IR) has been steadily building in salience in the 2010s and more drastically in the 2020s. The release of generative AI models, such as ChatGPT in November 2022, deepened existing international debates and expanded a (veritable) flurry of global, regional and national AI governance initiatives around AI ethics, responsible AI, AI risks, and AI safety. When IR first started paying attention to AI technologies in the early 2000s, such considerations were mainly restricted to questions of warfare and security, narrowly understood. In other words, scholars and commentators chiefly concerned themselves with technological developments in the military domain, especially in the form of autonomous weapon systems that, once activated, can ‘track, identify, and attack targets with violent force without further human intervention’.^1^

This initial focus has diversified along with the growing salience of AI technologies. After all, AI or related technologies such as automation and autonomy can be used to fulfil functions beyond those related to tactical–operational tail-end of the targeting process. The oft-cited ‘fighting at machine speed’ does not only refer to the potential of systems used in combat that could function without communication and control links, but also to how many militaries associate AI technologies with more effective, rapid decision-making when processing large amounts of data.^2^ AI technologies may therefore be used for various types of ‘assistance tasks’ in broader military decision-making processes (often reframed as ‘decision-support’) such as filtering data, selecting information, aggregating information across different datasets, identifying patterns, and drawing conclusions. Such decision-support systems can be used for descriptive, but also for predictive or prescriptive purposes.^3^ Further, a 2023 report published by the UN Institute for Disarmament Research has categorised at least six broad military applications of AI technologies: command and control; logistics; information management; casualty care; training; and cyber.^4^ It is important to be aware of this broader picture when thinking about AI in the context of IR.

**Figure:**
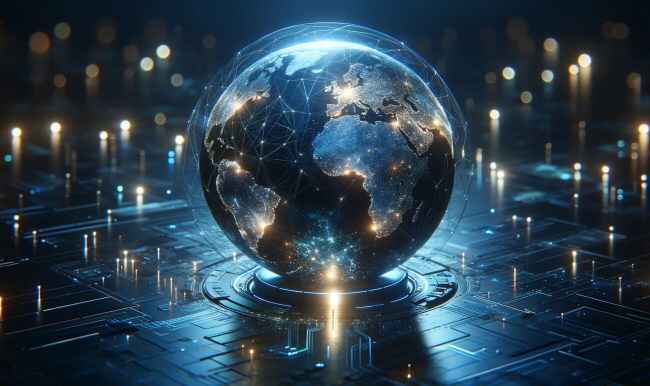
Emerging research avenues about AI and IR appear to take the thinking beyond the four established themes by engaging in novel analytical conceptualisations. *Generated by AI. Courtesy of Choi Poo / Alamy*

In the following, I offer my reading on how AI technologies have been studied in the discipline of IR. I structure this reading into two parts: first, I summarise the state of the art on AI in IR into four big themes: the balance of power; governance; disinformation; and ethics. Second, I identify three emerging research avenues that take our thinking about AI technologies and IR further: (re)conceptualising technology; beyond the AI arms race; and unpacking relevant actors. Across both of these sections, I pay particular attention to what scholars have to say about the need for conceptual innovation that understanding AI in IR triggers. In other words, does fully accounting for the significance of AI technologies require IR scholars to develop novel analytical frameworks or concepts? Scholars tend to always be interested in putting forward novel concepts. There are several dynamics fueling this trajectory, including publishing politics. But the impetus to develop new concepts goes beyond such an instrumental logic. Novel analytical concepts or concepts that are transferred to the study of AI in IR from other disciplines should, after all, allow us to make better sense of this development, to see things that we have not yet seen, and to therefore advance our collective understanding about the significance of AI.

First, however, I will address ‘AI’ as a term. Many scholars have expressed concerns around the hype associated with the term and how it needs to be demystified.^5^ I follow a broad understanding of AI based on a definition by a computer scientist: ‘the attempt to create machines or things that can do more than what is programmed into them’.^6^ Although AI often appears to be most closely associated with machine learning,^7^ that is, of course, the dominant rather than the only technique used. The AI systems that draw most public attention are large language models (LLMs) or forms of computer vision. Referring to something as ‘AI’ can also have the effect of distinguishing or distancing what is happening at the current moment from predecessor technologies that have been around for much longer, such as automation and autonomy.^8^ It is important to consider the historical trajectory of this technological development to situate and demystify the current hype.

## AI in IR: The State of the Art

IR scholarship has chiefly covered AI in four big themes: balance of power; governance; disinformation; and ethics. I proceed to describe the main contours of these, sometimes overlapping, themes, accompanied by reflecting on the extent to which scholarship in these themes largely builds on or extends existing theoretical–analytical ‘wisdom’ in IR.

### Balance of Power

The first theme is a strategic studies perspective on AI. Research questions turn around, for example, how becoming a leading state on AI may affect the balance of power and how the pursuit of AI in the context of renewed geopolitical competition between the great powers, especially China and the US, leads to security competition around AI, sometimes captured in the notion of an ‘AI arms race’.^9^ In other words, scholars examine the potential effect that the pursuit of, access to and employment of AI may have on current and future strategic stability.^10^

Such studies often start from the basic idea that AI is pivotal in thinking about the balance of power because it does not only affect military capabilities but also the extent to which states can project power both regionally and globally – in other words, how states can build and extend their spheres of influence.^11^ There is a connected notion that the greater your amount of power, fundamentally impacted by AI, the greater your potential to exercise influence in political, economic and military terms. This argument then leads to security competition around AI as an outcome.^12^ Because AI has the potential to affect the balance of power, AI will likely have an effect on strategic rivalries between the great powers and may lead to an overall increase in instability. Thus, there is a security competition around AI in broad terms but also in specific terms – because of the potential influence that AI technologies could have on military capabilities and military power specifically. There are many echoes of the security dilemma in this line of thinking, classically defined by Robert Jervis as ‘many of the means by which a state tries to increase its security decrease the security of others’.^13^

A further stream of scholarship in strategic studies, mostly coming from scholars interested in (military) innovation, is far more sceptical about the actual significance of AI for international security. Here, the arguments are either that AI is not that revolutionary or that it does not present as distinct military advantages as is argued elsewhere.^14^

As this brief review has already illustrated, conceptually, scholarship in strategic studies appears to work chiefly with established concepts – often drawing on structural realism, as well as broader strategic studies, and adding AI to that mix. Notably, this line of thinking follows instrumental and/or substantive/determinist perspectives on technology.^15^ The instrumental approach maintains that technologies are ‘neutral’ tools that actors, such as states, might use to attain their goals. However, these technologies are ultimately considered to be of lesser importance in terms of analytical value. The substantive or determinist approach examines how technologies follow an independent logic and development trajectory that actors cannot influence but rather only react to. Overall, scholarship in strategic studies does not appear to identify a significant need for new concepts specifically in relation to AI because established concepts appear to serve them well.

### Governance

The second theme is a growing conversation around AI governance, bringing together legal scholars, scholars of global governance and scholars of norms. Research questions cover how AI can be governed, persistent obstacles to AI governance at the international (and regional) level, and what happens in the absence of top-down forms of AI governance.^16^

There has been a veritable deluge of AI governance initiatives over the past 5–10 years and we appear to be getting to a point where almost every international and regional organisation has their own AI governance initiative. Some examples are the EU’s AI Act (2023),^17^ the African Union’s AI policy draft (2024),^18^ the OECD’s Recommendation on AI (2019),^19^ and the UN’s High-Level Advisory Body on AI (2023–ongoing).^20^ Some literature in this stream therefore focuses on summarising and categorising key AI governance initiatives.^21^ But as the number of governance initiatives is constantly increasing, it is hard to keep track.

What this development shows is a shared recognition that global governance of AI is needed. Despite their differences, there is an overall assessment among many states and intergovernmental organisations that current international law is not sufficient to address the challenges raised by AI technologies. There is less agreement over what kind of form AI governance should actually take. The AI governance initiatives that have so far resulted in outcomes have typically formulated declarations or lists of principles. In other words, they remain at the level of soft international law.

It is also notable that these lists of principles achieved at the international level tend to be relatively vague and ambiguous in nature. An example is the OECD’s Recommendation on AI, which includes robustness, security and safety as key AI governance principles, noting that ‘AI systems should be robust, secure, and safe throughout the entire lifecycle so that, in conditions of normal use, foreseeable use or misuse, or other adverse conditions, they function appropriately and do not pose unreasonable safety and/or security risks’.^22^ This principle leaves many questions open, for example, how do we distinguish between unreasonable and reasonable safety and security risks? Internationally agreed texts or even treaties often include such constructive ambiguity as a typical feature as the basis for agreeing on a shared text in the first place.^23^ But it does mean that there is considerable room for interpreting the text and implementing its measures. In the context of AI, this could be particularly detrimental for securing effective AI governance because the devil is really in the detail. While some of the established ethical AI principles therefore sound very good on paper – who would not want to support responsible AI? – how precisely they will be implemented and whether implementation across different political entities can be aligned is less clear.

While all of this is going on, we continue, by and large, to have an international governance gap on AI technologies. This will continue for as long as there is no significant movement towards some more comprehensive and operationalised form of international AI governance. In light of this, scholarship has also addressed the question of what happens in the absence of such a top-down governance set-up. Findings here point to how norms, broadly defined as understandings of appropriateness, are shaped by how actors design and use AI technologies, for example in weapon systems.^24^

Looking at the concepts used in this research theme, the picture is mixed, as scholars draw on established IR concepts, such as global governance and norms, but also turn towards novel conceptualisations not previously part of the IR canon to make sense of AI technologies, notably from science and technology studies (STS).

### Disinformation

A third theme of IR scholarship examines the cross-section of AI (and other digital) technologies and disinformation – ‘information that is deliberately false or misleading’.^25^ Literature centres around presenting concerted disinformation campaigns by foreign states, especially Russia, including in the context of hybrid forms of warfare combining cyber and conventional aspects.^26^ To a more limited extent, this literature also covers China^27^ as well as non-state actors as ‘a major threat to Western democracies and to the international institutions which they built’.^28^ Such a relationship is potentially shaped and elevated by AI because of how such technologies enable the spread of disinformation in ‘unprecedented breadth, width, depth’^29^ and speed across a large number of users.^30^ Furthermore, AI technologies may make it more difficult to distinguish disinformation from misinformation, material that does not deliberately intend to mislead.^31^

While media manipulation is not new, using AI to produce, for example, deep fakes has raised particular apprehension because the results are becoming more realistic and are quickly and inexpensively made, using publicly accessible software.^32^ Thus, even untrained users might potentially obtain the necessary software tools and use publicly available data to produce increasingly plausible counterfeit material. State opponents or politically motivated individuals may therefore disseminate fabricated footage of elected leaders or other public figures making incendiary comments or behaving inappropriately.^33^ This, some scholarship warns, could erode public trust, have a negative impact on public debate, or even tilt an election.^34^ However, other studies point out that measuring such an impact of technology-induced disinformation or manipulation of information is proving ‘nearly always impossible’.^35^

IR scholarship in this vein tends to be intradisciplinary in nature, drawing, for example, significantly on insights from media and communication studies. Much of the thinking draws on established concepts and arguments – including a certain degree of scepticism around the ‘unique’ departures that AI supposedly represents.

### Ethics

A fourth and final established theme turns around, broadly, ethical considerations and effects of AI technologies. This debate about AI ethics has some overlap with the previous governance theme. But there is also a more fundamental conversation about the ethics of using AI technologies in the context of IR and especially in relation to the military domain. Questions covered here ask what we should do with AI technologies, what kind of tasks should be shared with or ‘delegated’ to AI technologies, what risks the use of these technologies involve, and how we can control AI technologies. Concepts that have been used include different understandings of ethics, human dignity, as well as moral agency.^36^ A lot of scholarship falls within this theme and it is challenging to summarise because there is a big diversity of perspectives – not least because this conversation is not only held in relation to IR but much more broadly.

Generally, this fourth theme has not been the most prominent one in IR, specifically, but it is an important theme because it touches upon fundamental issues that matter when we think about the impact of AI. For example, there are instances of human–machine interaction across various political and social fields as AI technologies spread. But what and how does this change the exercise of agency,^37^ that is the ability to make choices and act?

In terms of the analytical concepts used, scholars interested in the ethical dimensions draw on a large variety of disciplines spanning across philosophy, applied ethics, political theory and psychology. Maybe more so than in some of the other key themes summarised, the emphasis appears to be decidedly on reflecting on the extent to which ‘old’ concepts still do sufficient analytical work.

## Emerging Research Avenues

Beyond the four established themes, three emerging research avenues appear to be taking thinking about AI technologies in IR further, typically by engaging in novel analytical conceptualisations.

### (Re)Conceptualising Technology

A first, fundamental emerging research avenue argues for reconceptualising technology. Scholars argue that fully accounting for the significance of AI technologies requires moving beyond instrumental and substantive/determinist accounts of technology. Rather, they argue that the development of technologies cannot be thought of as unfolding separately from political and social contexts, but rather as part of and within such contexts.^38^ In other words, technologies are social entities. This line of thinking comes from STS^39^ and has been gaining ground in IR, in general – but has gathered more momentum in the context of studying AI. Some of the research I have just summarised already uses these STS understandings of technology – for example my own contributions that look at how data and design choices can become sources of norms. But it is worthwhile drawing attention to (re)conceptualising technology as an emerging research avenue because IR appears to, for long, have had a technology problem.^40^ Technology is a significant ‘variable’ in strategic studies, as summarised, but it has not been adequately conceptualised. We can attribute a more comprehensive conceptualisation of technology in IR to the critical security studies agenda, which focuses on analysing the interplay between security, technology and culture.^41^ The emerging research avenue reconceptualising technology in IR’s AI research space notably connects directly to this tradition.

### Beyond the AI Arms Race

A second avenue of emerging research contests the AI arms race as the ‘inevitable outcome’ or the dominant frame of thinking about AI and IR. Some argue that ‘AI arms race’ is simply not the right term to use and favour terms such as AI competition or security competition over AI.^42^ Although the terminology is different, the analytical effect here is more or less the same.

Other scholars’ critique is more fundamental in nature as they are interested in the consequences of this framing having become one of the dominant understandings of AI in foreign affairs.^43^ This includes new research projects, such as a project on AI and geopolitics led by Verity Harding that will examine how the AI arms race frame increases tensions between ‘great powers’ and may lead to a governance race to the bottom.^44^ The argument here is that thinking about AI in IR primarily as an ‘arms race’ overshadows the potential for achieving progress through collaboration and via AI technologies, something that, for example, the annual ‘AI for Good Global Summit’ in Geneva seeks to champion.^45^ The AI arms race frame therefore has direct consequences in terms of how we consider approaching the spread of AI technologies in IR. As an analytical frame, the AI arms race also has consequences for who we consider as the main actors. It is a way of thinking about AI technologies that privileges powerful state actors, in particular the ‘great powers’. This is exactly what the third emerging avenue of research writes against.

### Unpacking Relevant Actors

A third emerging avenue of research argues that fully appreciating the significance of AI technologies in IR should lead us beyond just looking at powerful states. Two sets of actors receive more attention here: tech companies and the Global South.

The role that tech companies play in the context of AI technologies is hugely significant and the subject of a number of emerging research endeavours focusing, inter alia, on Big Tech.^46^ The theme of investigating private authority in global governance is not new in IR but started as an analytical concern in the late 1990s.^47^ What appears to be new, however, is the status of such tech companies as contributors to shaping international governance of AI in explicit and implicit ways.^48^ Explicitly, we see that many state actors or international organisations seek out tech company representatives to participate in AI governance conversations. This can take various forms, for instance as part of ‘expert advisory boards’ or as direct participants at AI governance events, such as the UK’s AI Safety Summit.^49^ There is a standing assumption that representatives of tech companies have the right kind of expertise to appropriately address the challenges raised by AI technologies and that they provide policymakers with all the necessary understanding and information needed. This is a remarkable move because it gives such tech companies a significant platform for shaping potential governance outputs. It also typically privileges expertise associated with tech companies over all other kinds of expertise that would be needed to comprehensively understand the challenges raised by AI technologies. But there are also more implicit ways in which tech companies shape AI governance. As long as we have no effective international top-down governance framework, those who design and use AI technologies shape de facto norms governing how AI technologies should be used.^50^

There are at least two different analytical ways of capturing the influence of tech companies. A first mode studies how such tech companies have come to be attributed with expert status in the conversations around AI. This leads to bigger question around who has the right expertise on matters of AI governance, the politics of attributing that expertise, and whether we need to rethink these dynamics. A second mode is to consider the material power capacities that are at the background of these conversations – that is to say, access to infrastructure and data. This line of research is not that present in IR yet but could be usefully integrated in an international political economy perspective.

A second group of actors that receives more attention is the Global South, broadly understood. A sole focus on the great powers omits attention to the majority of the world’s countries who are just as affected by the spread of AI technologies and its consequences. Accounting for these Global South actors is important for various reasons. First of all, we find ourselves in a situation where the international order is changing from a previously dominant rules-based international order to something new. Some scholars already argue that we no longer find ourselves in a singular version of the international order but in a situation where there are multiple orders, a multi-order world with different ‘leading states’ per order, such as a US-led order, a Chinese-led order and a Russian-led order.^51^ We may contest this particular framing, but there is an overall agreement that international order is changing and the direction this will take is not entirely clear. This makes the Global South so important because they may consider the pursuits attached to one of these (or other) orders more appropriate than the other. These choices will shape IR. And AI technologies appear to play a big role therein because there is a significant danger of AI governance regimes simply replicating exclusive governance regimes of the past.

## Conclusion

In sum, the study of AI in IR already has a, possibly longer than expected, track record. But while much, if not all, of the work in the established research themes appears to fit to a considerable degree within the well-traded analytical confines of IR as a discipline, we also see an emerging number of research themes that directly push this status quo into different and novel directions. This is frequently animated by understanding AI as socio-technical systems and thereby taking technology seriously as an analytical factor in IR. But we also see scholarship writing against what can be described as a strong return of rationalist–structural–realist approaches to the study of IR in the form of a geopolitical competition around AI. The main reference point for this research is rather a critique of how this rationalist research tends to privilege particular state actors, and turns around questioning whether such an analytical dominance can be justified as a theoretical lens to appreciate the significance of AI technologies at what may constitute a pivotal disciplinary moment. ◼

## Notes

1 ICRC, ‘ICRC Position on Autonomous Weapon Systems’, 12 May 2021, <https://www.icrc.org/en/document/icrc-position-autonomous-weapon-systems>, accessed 10 June 2024.

2 Michael C Horowitz, ‘When Speed Kills: Lethal Autonomous Weapon Systems, Deterrence and Stability’, *Journal of Strategic Studies* (Vol. 42, No. 6, September 2019), p. 769; Frank Sauer, ‘The Military Rationale for AI’, in Niklas Schörnig and Thomas Reinhold (eds), *Armament, Arms Control and Artificial Intelligence* (Cham: Springer Nature, 2022), pp. 31–33.

3 Geneva Academy and ICRC, ‘Artificial Intelligence and Related Technologies in Military Decision-Making on the Use of Force in Armed Conflicts: Current Developments and Potential Implications’, March 2024, p. 9.

4 Sarah Grand-Clément, ‘Artificial Intelligence Beyond Weapons: Application and Impact of AI in the Military Domain’, United Nations Institute for Disarmament Research, 2023.

5 Meredith Whittaker, ‘The Steep Cost of Capture’, *Interactions* (Vol. 28, No. 6, 2021), pp. 50–55; Emily Tucker, ‘Artifice and Intelligence’, Tech Policy Press, 17 March 2022, <https://techpolicy.press/artifice-and-intelligence/>, accessed 12 June 2023.

6 Timnit Gebru, ‘Don’t Fall for the AI Hype’, *Tech Won’t Save Us* Podcast with Paris Marx, 19 January 2023, <https://techwontsave.us/episode/151_dont_fall_for_the_ai_hype_w_timnit_gebru>, accessed 1 September 2023.

7 For example, see Stephane J Baele et al., ‘AI IR: Charting International Relations in the Age of Artificial Intelligence’, *International Studies Review* (Vol. 26, No. 2, June 2024), p. 9; Horowitz, ‘When Speed Kills’, p. 769.

8 Ingvild Bode et al., ‘Prospects for the Global Governance of Autonomous Weapons: Comparing Chinese, Russian, and US Practices’, *Ethics and Information Technology* (Vol. 25, No. 1, March 2023), p. 5.

9 Kareem Ayoub and Kenneth Payne, ‘Strategy in the Age of Artificial Intelligence’, *Journal of Strategic Studies* (Vol. 39, No. 5–6, September 2016), pp. 793–819; Lance Y Hunter et al., ‘The Military Application of Artificial Intelligence Technology in the United States, China, and Russia and the Implications for Global Security’, *Defense & Security Analysis* (Vol. 39, No. 2, April 2023), pp. 207–32.

10 Jürgen Altmann and Frank Sauer, ‘Autonomous Weapon Systems and Strategic Stability’, *Survival* (Vol. 59, No. 5, September 2017), pp. 117–42; James Johnson, ‘Artificial Intelligence, Drone Swarming and Escalation Risks in Future Warfare’, *RUSI Journal* (Vol. 165, No. 2, February 2020), pp. 26–36; James Johnson, *Artificial Intelligence and the Future of Warfare: The USA, China, and Strategic Stability* (Manchester: Manchester University Press, 2021); Horowitz, ‘When Speed Kills’.

11 Hunter et al., ‘The Military Application of Artificial Intelligence Technology in the United States, China, and Russia and the Implications for Global Security’, p. 209.

12 Paul Scharre, ‘Debunking the AI Arms Race Theory’, *Texas National Security Review* (Vol. 4, No. 3, 2021), pp. 121–32.

13 Robert Jervis, ‘Cooperation under the Security Dilemma’, *World Politics* (Vol. 30, No. 2, 1978), p. 169.

14 Antonio Calcara et al., ‘Why Drones Have Not Revolutionized War: The Enduring Hider-Finder Competition in Air Warfare’, *International Security* (Vol. 46, No. 4, April 2022), pp. 130–71.

15 Andrew Feenberg, *Critical Theory of Technology* (Oxford: Oxford University Press, 1991); Richard Wyn Jones, *Security, Strategy and Critical Theory* (London: Lynne Rienner, 1999).

16 Michael Veale, Kira Matus and Robert Gorwa, ‘AI and Global Governance: Modalities, Rationales, Tensions’, *Annual Review of Law and Social Science* (Vol. 19, 2023), pp. 1–30; Bode et al., ‘Prospects for the Global Governance of Autonomous Weapons’; Jonas Tallberg et al., ‘The Global Governance of Artificial Intelligence: Next Steps for Empirical and Normative Research’, *International Studies Review* (Vol. 25, No. 3, September 2023); Denise Garcia, *Common Good Governance in the Age of Military Artificial Intelligence* (Oxford: Oxford University Press, 2023); Matthijs M Maas, ‘Innovation-Proof Governance for Military AI? How I Learned to Stop Worrying and Love the Bot’, *Journal of International Humanitarian Legal Studies* (Vol. 10, No. 1, 2019), pp. 129–57.

17 European Parliament, ‘EU AI Act: First Regulation on Artificial Intelligence’, 8 June 2023, <https://www.europarl.europa.eu/topics/en/article/20230601STO93804/eu-ai-act-first-regulation-on-artificial-intelligence>, accessed 13 June 2024.

18 African Union, ‘African Ministers Adopt Landmark Continental Artificial Intelligence Strategy, African Digital Compact to drive Africa’s Development and Inclusive Growth’, press release, 17 June 2024 , <https://au.int/en/pressreleases/20240617/african-ministers-adopt-landmark-continental-artificial-intelligence-strategy>, accessed 18 June 2024.

19 OECD, ‘Recommendation of the Council on Artificial Intelligence’, OECD Legal Instruments, 22 May 2019, <https://legalinstruments.oecd.org/en/instruments/OECD-LEGAL-0449>, accessed 13 June 2024.

20 UN, ‘High-Level Advisory Body on Artificial Intelligence’, <https://www.un.org/techenvoy/ai-advisory-body>, accessed 13 June 2024.

21 Allan Dafoe, ‘AI Governance: A Research Agenda’, Centre for the Governance of AI, University of Oxford, 2018, <https://www.fhi.ox.ac.uk/wp-content/uploads/GovAI-Agenda.pdf>, accessed 13 June 2024; Matthijs Maas and José Jaime Villalobos, ‘International AI Institutions: A Literature Review of Models, Examples, and Proposals’, AI Foundations Report, Legal Priorities Project, September 2023; Veale, Matus and Gorwa, ‘AI and Global Governance’; Tallberg et al., ’The Global Governance of Artificial Intelligence’.

22 OECD, ‘Recommendation of the Council on Artificial Intelligence’.

23 Michael Byers, ‘Still Agreeing to Disagree: International Security and Constructive Ambiguity’, *Journal on the Use of Force and International Law* (Vol. 8, No. 1, 2021), pp. 91–114; Antoine Rayroux, ‘Speaking EU Defence at Home: Contentious Discourses and Constructive Ambiguity’, *Cooperation and Conflict* (Vol. 49, No. 3, 2014), pp. 386–405.

24 Ingvild Bode, ‘Practice-Based and Public-Deliberative Normativity: Retaining Human Control over the Use of Force’, *European Journal of International Relations* (Vol. 29, No. 4, 2023), pp. 990–1016; Bode et al., ‘Prospects for the Global Governance of Autonomous Weapons’.

25 Caroline Jack, ‘Lexicon of Lies: Terms for Problematic Information’, Data and Society Research Institute, September 2017, p. 3, <https://datasociety.net/pubs/oh/DataAndSociety_LexiconofLies.pdf>, accessed 13 June 2024.

26 Agnieszka Legucka and Robert Kupiecki, *Disinformation, Narratives and Memory Politics in Russia and Belarus*, 1^st^ edition (Abingdon: Routledge, 2022); Rod Thornton and Marina Miron, ‘Towards the “Third Revolution in Military Affairs”: The Russian Military’s Use of AI-Enabled Cyber Warfare’, *RUSI Journal* (Vol. 165, No. 3, April 2020), pp. 12–21.

27 David M Beskow and Kathleen M Carley, ‘Characterization and Comparison of Russian and Chinese Disinformation Campaigns’, in Kai Shu et al. (eds), *Disinformation, Misinformation, and Fake News in Social Media: Emerging Research Challenges and Opportunities* (Cham: Springer International Publishing, 2020), pp. 63–81.

28 André W M Gerrits, ‘Disinformation in International Relations: How Important Is It?’, *Security and Human Rights* (Vol. 29, 2018), p. 6.

29 *Ibid.*, p. 19.

30 *Ibid.*, p. 19; Steven Feldstein, ‘The Consequences of Generative AI for Democracy, Governance and War’, *Survival* (Vol. 65, No. 5, 2023), pp. 117–42.

31 xe "citationID"Jack, ‘Lexicon of Lies’, p. 2.

32 Robert Chesney and Danielle Citron, ‘Deepfakes and the New Disinformation War: The Coming Age of Post-Truth Geopolitics’, *Foreign Affairs* (Vol. 98, No. 1, 2019). p. 147.

33 Feldstein, ‘The Consequences of Generative AI for Democracy, Governance and War’.

34 Thomas Paterson and Lauren Hanley, ‘Political Warfare in the Digital Age: Cyber Subversion, Information Operations and “Deep Fakes”’, *Australian Journal of International Affairs* (Vol. 74, No. 4, July 2020), pp. 439–54.

35 Gerrits, ‘Disinformation in International Relations’, p. 8.

36 Elke Schwarz, *Death Machines: The Ethics of Violent Technologies* (Manchester: Manchester University Press, 2019); Birigit Schippers, ‘Autonomous Weapons Systems and Ethics in International Relations’, in Birigit Schippers (ed.), *The Routledge Handbook to Rethinking Ethics in International Relations (London: Routledge, 2020); Peter Asaro, ‘Autonomous Weapons and the Ethics of Artificial Intelligence’, in S Matthew Liao (ed.), The Ethics of Artificial Intelligence* (Oxford: Oxford University Press, 2020), pp. 1–20; Alexander Blanchard, Christopher Thomas and Mariarosaria Taddeo, ‘Ethical Governance of Artificial Intelligence for Defence: Normative Tradeoffs for Principle to Practice Guidance’, *AI & Society*, 19 February 2024; Neil Renic and Elke Schwarz, ‘Crimes of Dispassion: Autonomous Weapons and the Moral Challenge of Systematic Killing’, *Ethics & International Affairs* (Vol. 37, No. 3, Fall 2023), pp. 321–43.

37 Andreas Tsamados, Luciano Floridi and Mariarosaria Taddeo, ‘Human Control of AI Systems: From Supervision to Teaming’, *AI and Ethics*, 28 May 2024, https://doi.org/10.1007/s43681-024-00489-4; Ingvild Bode and Anna Nadibaidze, ‘Symposium on Military AI and the Law of Armed Conflict: Human-machine Interaction in the Military Domain and the Responsible AI Framework’, Opinio Juris, 4 April 2024, <https://opiniojuris.org/2024/04/04/symposium-on-military-ai-and-the-law-of-armed-conflict-human-machine-interaction-in-the-military-domain-and-the-responsible-ai-framework/>, accessed 13 June 2024.

38 Marijn Hoijtink and Matthias Leese (eds), *Technology and Agency in International Relations* (London: Routledge, 2019); Rocco Bellanova, Katja Lindskov Jakobsen and Linda Monsees, ‘Taking the Trouble: Science, Technology and Security Studies’, *Critical Studies on Security* (Vol. 8, No. 2, 2020), pp. 87–100; Rocco Bellanova et al., ‘Toward a Critique of Algorithmic Violence’, *International Political Sociology* (Vol. 15, No. 1, March 2021), pp. 121–50.

39 Wiebe E Bijker and John Law (eds), *Shaping Technology/Building Society: Studies in Sociotechnical Change* (Cambridge, MA: MIT Press, 2010); Ursula Franklin, *The Real World of Technology* (Berkeley, CA: House of Anansi Press, 1999).

40 My thanks go to Tom Watts for coining this apt expression.

41 Columba Peoples, *Justifying Ballistic Missile Defence: Technology, Security and Culture* (Cambridge: Cambridge University Press, 2010), p. 2.

42 Scharre, ‘Debunking the AI Arms Race Theory’.

43 Heather M Roff, ‘The Frame Problem: The AI “Arms Race” isn’t One’, Bulletin of the Atomic Scientists, 29 April 2019, <https://thebulletin.org/2019/04/the-frame-problem-the-ai-arms-race-isnt-one/>, accessed 13 June 2024.

44 Verity Harding, ‘What Are the Geopolitical Implications of AI?’, University of Cambridge, 1 November 2023, <https://ai.cam.ac.uk/blog/verity-harding-generative-ai>, accessed 13 June 2024.

45 AI for Good, ’AI for Good Global Summit’, ITU, <https://aiforgood.itu.int/summit24/>, accessed 13 June 2024.

46 Marijn Hoijtink, ‘“Prototype Warfare”: Innovation, Optimisation, and the Experimental Way of Warfare’, *European Journal of International Security* (Vol. 7, No. 3, August 2022), pp. 322–36; Marijn Hoijtink and Anneroos Planqué-van Hardeveld, ‘Machine Learning and the Platformization of the Military: A Study of Google’s Machine Learning Platform TensorFlow’, *International Political Sociology* (Vol. 16, No. 2, June 2022).

47 A Claire Cutler, Virginia Haufler and Tony Porter (eds), *Private Authority and International Affairs* (Albany, NY: State University of New York Press, 1999); Rodney Bruce Hall and Thomas J Biersteker, ‘The Emergence of Private Authority in the International System’, in Rodney Bruce Hall and Thomas J Biersteker (eds), *The Emergence of Private Authority in Global Governance* (Cambridge: Cambridge University Press, 2002), pp. 3–22; Anna Leander, ‘Practices (Re)Producing Orders: Understanding the Role of Business in Global Security Governance’, in Anna Leander and Morten Ougaard (eds), *Business and Global Governance* (Abingdon: Routledge, 2010), pp. 57–77.

48 Ingvild Bode and Hendrik Huelss, ‘Constructing Expertise: The Front- and Back-Door Regulation of AI’s Military Applications in the European Union’, *Journal of European Public Policy* (Vol. 30, No. 7, February 2023), pp. 1230–54; Anke Sophia Obendiek and Timo Seidl, ‘The (False) Promise of Solutionism: Ideational Business Power and the Construction of Epistemic Authority in Digital Security Governance’, *Journal of European Public Policy* (Vol. 30, No. 7, July 2023), pp. 1305–29.

49 Department for Science, Innovation & Technology, ‘AI Safety Summit: Confirmed Attendees (Governments and Organisations)’, updated 31 October 2023, <https://www.gov.uk/government/publications/ai-safety-summit-introduction/ai-safety-summit-confirmed-governments-and-organisations>, accessed 13 June 2024.

50 Ingvild Bode and Hendrik Huelss, ‘Artificial Intelligence Technologies and Practical Normativity/Normality: Investigating Practices beyond the Public Space’, *Open Research Europe* (March 2024), DOI:10.12688/openreseurope.16536.2.

51 Trine Flockhart, ‘The Coming Multi-Order World’, *Contemporary Security Policy* (Vol. 37, No. 1, 2016), pp. 3–30.

